# Pericytes in Microvessels: From “Mural” Function to Brain and Retina Regeneration

**DOI:** 10.3390/ijms20246351

**Published:** 2019-12-17

**Authors:** Nunzia Caporarello, Floriana D’Angeli, Maria Teresa Cambria, Saverio Candido, Cesarina Giallongo, Mario Salmeri, Cinzia Lombardo, Anna Longo, Giovanni Giurdanella, Carmelina Daniela Anfuso, Gabriella Lupo

**Affiliations:** 1 Department of Physiology & Biomedical Engineering, Mayo Clinic, Rochester, MN 55905, USA; Caporarello.Nunzia@mayo.edu; 2 Section of Medical Biochemistry, Department of Biomedical and Biotechnological Sciences, School of Medicine, University of Catania, 95123 Catania, Italy; floriana.dangeli@hotmail.it (F.D.); cambrimt@unict.it (M.T.C.); longo.anna@hotmail.it (A.L.); g.giurdanella@unict.it (G.G.); 3 Section of General and Clinical Pathology and Oncology, Department of Biomedical and Biotechnological Sciences, School of Medicine, University of Catania, 95123 Catania, Italy; saverio.candido@unict.it; 4 Section of Haematology, Department of General Surgery and Medical-Surgical Specialties, University of Catania, 95123 Catania, Italy; cesarinagiallongo@yahoo.it; 5 Section of Microbiology, Department of Biomedical and Biotechnological Sciences, School of Medicine, University of Catania, 95123 Catania, Italy; msalmeri@unict.it (M.S.); cinzialombardo@hotmail.com (C.L.)

**Keywords:** Pericytes, microvessels, MSC features, brain and retina repair, stroke, brain tumor, diabetic retinopathy, ocular diseases

## Abstract

Pericytes are branched cells located in the wall of capillary blood vessels that are found throughout the body, embedded within the microvascular basement membrane and wrapping endothelial cells, with which they establish a strong physical contact. Pericytes regulate angiogenesis, vessel stabilization, and contribute to the formation of both the blood-brain and blood-retina barriers by Angiopoietin-1/Tie-2, platelet derived growth factor (PDGF) and transforming growth factor (TGF) signaling pathways, regulating pericyte-endothelial cell communication. Human pericytes that have been cultured for a long period give rise to multilineage progenitor cells and exhibit mesenchymal stem cell (MSC) features. We focused our attention on the roles of pericytes in brain and ocular diseases. In particular, pericyte involvement in brain ischemia, brain tumors, diabetic retinopathy, and uveal melanoma is described. Several molecules, such as adenosine and nitric oxide, are responsible for pericyte shrinkage during ischemia-reperfusion. Anti-inflammatory molecules, such as IL-10, TGFβ, and MHC-II, which are increased in glioblastoma-activated pericytes, are responsible for tumor growth. As regards the eye, pericytes play a role not only in ocular vessel stabilization, but also as a stem cell niche that contributes to regenerative processes in diabetic retinopathy. Moreover, pericytes participate in melanoma cell extravasation and the genetic ablation of the PDGF receptor reduces the number of pericytes and aberrant tumor microvessel formation with important implications for therapy efficacy. Thanks to their MSC features, pericytes could be considered excellent candidates to promote nervous tissue repair and for regenerative medicine.

## 1. Introduction

Located on the abluminal surface of capillary blood vessels throughout the body, pericytes are specialized cells with a pivotal role in vascular homoeostasis [1]. Pericytes were described for the first time by Rouget in 1879, and for this reason they were first named “Rouget cells”. Because of their location on the outer surface of blood capillaries and their close interaction with the underlying endothelial cells (ECs), with which they share the basement membrane, in 1923 Zimmermann renamed them “pericytes” (peri: around; cyte: cell) [2]. During embryonic developmental stages, brain and retinal pericytes arise from the neuroectodermal neural crest, whereas other similar cells (the ones of the trunk of the body) arise from the mesoderm leaflet [3]. These cells have a prominent nucleus and a small cytoplasmic volume, and present distinct morphology in different organs. They can be fat, elongated or stellate with multiple cytoplasmatic processes, as observed in the central nervous system (CNS) phenotype, or rounded and compact (typical of kidney glomerulus mesangial cells) [4].

Pericytes presenting processes from their cell body are in contact with more than one EC. Direct physical connection between pericytes and endothelial cells is provided by gap junctions, which enable the exchange of small molecules and ions. Pericytes are anchored to endothelial cells through adhesion plaques, while peg and socket contacts allow the cells to penetrate into discontinuities in the basement membrane of vessels in contact with each other [5]. These junction complexes allow the transmission of mechanical contractile forces from pericytes to endothelial cells, and include cell-adhesion molecules, N-cadherin/β-catenin based adherent junctions, and extracellular matrix protein, i.e., fibronectin [6]. 

Moreover, the ratio of pericytes to endothelial cells is different, depending on the vascular districts. It varies from 1:1 in the retina and CNS to 1:10 in the skin and lung, and 1:100 in striated muscle [7], and also depends on the function of the specific tissue. Pericyte coverage of the abluminal vessel area is maximal in the CNS because of the contribution of pericytes in the anatomical composition of both the blood-brain barrier and the blood-retina barrier (BBB and BRB) [8].

Within the microcapillary stream, pericytes exert different functions: (1) angiogenesis modulation and vessel stabilization; (2) blood flow regulation; and (3) anatomical stabilization of the BBB and BRB. Moreover, pericytes have mesenchymal stem cell features [9,10,11,12]. 

The behavior of pericytes following brain ischemia, tumor and ocular diseases is described in this review. Several molecules and related signaling pathways are involved in pericyte response to pathologic processes. Among these are the following: NO/guanylate cyclase and PDGF-B mediating pericyte relaxation, platelet derived growth factor B (PDGF-B)/platelet derived growth factor β receptor (PDGFR- β) axis, restoring ischemic tissue through pericyte recruitment, and matrix metalloproteinase-9 (MMP-9), released by pericytes to increase microvessel permeability [5]. Moreover, pericytes secrete large amounts of TGF-β, which enhance the expression of perycites vascular endothelial growth factor receptor 1 (VEGFR-1) and vascular endothelial growth factor (VEGF). The latter induces the up-regulation of Ang-1/Tie-2, acting in an autocrine way, and of endothelial Ang-2 expression, mediating angiogenesis. In this review, the pericyte regenerative potential in brain ischemia reperfusion and tumors is reported. Moreover, as pericytes are particularly abundant in retinal microvessels, their role in ocular diseases, such as diabetic retinopathy and uveal melanoma, is examined. Because of the derivation of pericytes from mesenchymal progenitor cells, they are considered excellent candidates for ocular repair processes.

The plasticity of pericytes, which are capable of differentiating into different cell types, makes them very similar to MSCs. However, a distinction has to be made between pericytes and MSCs from different tissues, in particular, bone marrow derived MSCs (BM-MSCs). Indeed, it has been demonstrated that the two cell types have different effects on wound healing processes: the application of BM-MSCs to the wound induces an acceleration of wound healing processes, whereas the application of pericytes does not have the same effect, slowing wound healing and inducing a fibrotic process [3]. In the light of what has been mentioned above, this review analyzes the state of the art of the research on the use of pericytes in the treatment of brain and ocular diseases.

## 2. Pericytes: Canonical and New Functions

### 2.1. Angiogenesis Modulation and Vessel Stabilization 

Pericytes play a pivotal role in angiogenesis and contribute to vessel formation, remodeling and stabilization [6]. Pericytes and endothelial cells are in close physical contact, and it is well known that many soluble factors and signaling pathways are fundamental for mutual intercellular communication. Among these, platelet derived growth factor B (PDGF-B)/platelet derived growth factor β receptor (PDGFR-β) signaling pathway is of primary importance because of its involvement in pericyte proliferation, survival, and attachment [13]. Indeed, during the early phases of angiogenesis, PDGF-B, secreted by sprouting endothelial cells, binds to the pericyte-specific receptor PDGFR-β, and this event results in the recruitment and adhesion of pericytes to endothelial cells. When this pathway is defective, the lack of pericyte coverage leads to endothelial hyperplasia, aberrant vasculature and microaneurysms [14]. Moreover, the genetic deletion of both PDGF-B and PDGFR-β in mice leads to perinatal death due to vascular dysfunction [15,16], and the ablation of endothelial-derived PDGF-B causes the reduction of retinal pericyte coverage, resulting in the change of capillary and venous diameter, microaneurysms, and proliferative retinopathy [17], thus confirming the key role played by pericytes in angiogenesis and vessel stabilization. However, in *pdgf-B* and *pdgfr-β* knockout mouse embryos, the lack of pericytes is almost total in the skin, heart, brain, kidney and lung, whereas in the liver the number of pericytes seems to be univariate [18], suggesting that other molecules are involved in pericyte recruitment.

Pericyte-endothelial cell communication is also regulated by the angiopoietin 1 (Ang-1)/Tie-2 signalling pathway. It has been proposed that the Ang-1/Tie-2 paracrine loop has reciprocal tendencies in comparison with PDGF-B/PDGFR-β and may mediate endothelial maturation and stability [19]. Although the conditional knockout of *ang-1* showed that this gene is not required for pericyte recruitment [20], it has been demonstrated that Ang-1, secreted by pericytes, is necessary for new vessel formation. In this regard, Ang-1 overexpression results in an increased vascular remodeling from immature to mature vessels [21]. Angiopoietin 2 (Ang-2) binds the receptor of Ang-1 (Tie-2) with the same affinity as angiopoietin 1 and it has been reported that it inhibits receptor phosphorylation induced by Ang-1, thus acting as an antagonist [22]. 

Another signaling pathway involved in both pericyte and endothelial cell proliferation, differentiation and survival is mediated by the transforming growth factor-β (TGF-β). There are two distinct TGF-β type-I receptors and one TGF-β type-II receptor, which are serine/threonine kinase receptors, expressed by both cell types, thus suggesting the complexity of this pathway. The TGF-β signal is transduced from the membrane to the nucleus via Smad 2/3 and Smad 4 [23]. The addition of the anti-TGF-β neutralizing antibody to endothelial cell/pericyte co-cultures is able to block pericyte-mediated endothelial growth inhibition, demonstrating a strong correlation between the signaling pathway triggered by TGF-β of pericytal origin and the modulatory effect on pericyte survival [24]. The activation of TGF-β leads to the inhibition of endothelial cell proliferation and migration as well as the differentiation of perivascular cells (i.e., mesenchymal stem cells and neural crest cells) into pericytes [5]. The prominent role of TGF-β in the maintenance of a correct vasculature is highlighted by studies conducted in mice lacking TGF-β type-I receptors, which showed an abnormal angiogenesis [25]. 

As perivascular cells differentiate, they begin to express VEGF, which is expressed in response to hypoxia. The isoform produced by perivascular cells is most commonly VEGF-A165, which binds to heparin sulfate proteoglycans on the cell surface where it remains associated with the cell [26]. The local release of VEGF is important in promoting endothelial cell survival and stabilizing newly formed vessels. However, the VEGF concentration does not encourage endothelial cell migration as occurs when more soluble isoforms are released [26,27]. Pericytes also have, on the cell surface, VEGFR-1, which binds VEGF and sequesters it from VEGFR-2 on the endothelial cells, therefore preventing the initiation of angiogenesis in mature, quiescent vessels [28]. 

VEGFR-2 activation is transduced mainly through the phosphorylation of several tyrosines such as the selective tyrosine Y951 (in the kinase insert domain) and tyrosines Y1175 and Y1214 (in the carboxy-terminal domain), mediating endothelial permeability and proliferation. RAS/RAF/ERK/MAPK are the traditional pathways that are activated downstream [29]. Tyrosine kinase inhibitors, by inhibiting the VEGFR-2 pathway, are effective in treating various cancer types [11]. 

### 2.2. Blood Flow Regulation

Pericytes are able to regulate the perfusion of fluids and cells thanks to their high level of α-smooth muscle actin, similar to the smooth muscle cells of larger vessels, which confers contractile ability to them [30]. The contractile function in pericytes was also highlighted by the identification of contractile proteins such as tropomyosin and myosin. Furthermore, pericytes express both cholinergic and adrenergic receptors (α-2 and β-2). Specifically, α-2 response leads to contraction, whereas β-2 stimulation causes pericyte relaxation [8]. An in vitro study showed that membrane permeabilization and the following administration of adenosine triphosphate (ATP) caused selective contraction of pericytes but not of other cells (i.e., endothelial and epithelial cells) under the same conditions, thus confirming the contractile properties of pericytes [31]. Capillary pericytes actively contribute to the fine tuning of blood flow through contractile responses to local metabolic needs, i.e., high levels of carbon dioxide induce pericyte relaxation (metabolic auto-regulation of blood flow) [32]. 

Contraction ability is highly important both in physiological and in pathological conditions. Indeed, pericyte contractility is fundamental for the regulation of blood flow in microvessels during traumatic brain injury [33], ischemia [34], and in physiological neurovascular coupling [35]. Under hypoxic conditions, the contraction of pericytes increases. In this event, pericyte constriction may be a cause of the long-lasting decrease of cerebral blood flow that occurs when an obstructed artery is opened up after a stroke [36].

Neurovascular coupling controls cerebral blood flow by signaling pathways that cause dilation of capillaries and arterioles. It has been found that capillary dilation occurs following the effect on pericytes of E2 prostaglandins, released by astrocytes, whereas arteriolar dilation occurs following the effect of nitric oxide (NO) released by inter-neurons. Furthermore, the dilation of capillaries by pericytes occurs faster than that of arterioles and offers a greater increase in blood flow [37]. Moreover, the effect of NO, released by the neurons of the granular layer, is two-fold, in that, in addition to causing vasodilation, it inhibits the synthesis of the powerful vasoconstrictor 20-Hydroxyeicosatetraenoic acid (20-HETE), which is produced in pericytes by the metabolism of arachidonic acid [38]. On the other hand, it has also been demonstrated, by analyzing the cellular components of the microvascular contractile apparatus, that the occlusion of blood vessels during stroke is not caused by pericytes but rather by smooth muscle cells. Pericytes, therefore, would seem to play a role in coordinating the signaling pathways between the neural and vascular compartments [39]. The differences found by researchers in pericyte functions, both in physiological and pathological conditions, could depend on their position on the vascular bed. Thus, pericytes that are found at the ends of capillaries and that contain α-smooth muscle actin (α-SMA) could physiologically regulate blood flow and be responsible for capillary restriction during ischemia. Pericytes in the central part of the capillary bed, which contain less α-SMA, would be physiologically responsible for maintaining the characteristics of the BBB and would cause BBB breakdown during ischemia. Finally, pericytes at the end of the vascular bed physiologically regulate the passage of immune cells from the blood to brain tissue and the noticeable increase of these cells during ischemia is due precisely to the lack of control by pericytes [39]. Being able to distinguish the three pericytes classes would make it possible to identify precise therapeutic targets to limit the damage caused by various brain pathologies.

### 2.3. Anatomical Stabilization of the BBB and BRB 

As is well known, the normal physiology of nervous tissue (CNS and retina) requires extremely rigorous control and protection of the neuropil, which is carried out by the BBB and BRB. Both barriers protect the neuronal milieu from harmful substances (e.g*.,* toxins, drugs) and microorganisms, and regulate the paracellular flow between cells and transendothelial fluid transport, ensuring an optimal chemical environment for synapses and neurotransmission [23]. Endothelial cells have always been considered the most characteristic cellular components, both because of their position (in contact with the bloodstream) and the above mentioned strict metabolic control. Pericytes have received much less attention than the other components of the microvascular bed; these cells are now considered fundamental constituents of both the BBB and BRB. The particular tightness of these two barriers is due to both tight and adherent junctions between endothelial cells; studies have shown that pericyte coverage is essential to form anatomically mature barriers and to maintain their function and tissue homeostasis, together with the endothelium, neuronal and glial cells, in a complex system named “neurovascular unit” [23]. An imbalance in pericyte coverage has been described in different CNS diseases [40], such as Alzheimer’s disease [41], diabetic retinopathy [42,43], neonatal intraventricular hemorrhage [44], and amyotrophic lateral sclerosis [45]. Pericyte loss results in BBB breakdown and the increase of permeability, accumulation of plasma-derived proteins and subsequent neuronal degenerative alterations [46]. In an in vitro model of a bacterial infection of the BBB, on the basis of co-culturing endothelial cells and pericytes, *E. coli* invasion induced pericyte loss, and consequently a TEER decrease and BBB permeability increase [47]. 

Preclinical data have shown that pericytes play an important “modulating” role in the initial stages of endothelial sprouting driven by a brain tumor [48]. For example, the new vessels associated with glioblastoma are characterized by the absence of pericytes and the reduction of VEGF release induces their recession [49]. As a result of this, abnormal pericyte integration into the capillary wall, associated with deficient coverage, could be partly responsible for vessel abnormalities that contribute to disease, bacterial infection from the blood to nervous tissue and metastasis.

Pericyte coverage is crucial during embryogenesis, in fact, in PDGFR-β mouse mutants, the permeability through the BBB dramatically increases in the CNS [50]. In PDGF-B transgenic mice, the loss of pericyte coverage in the BRB is associated with reduced neuronal layers and a folding photoreceptor layer, signs that can be compared to diabetic retinopathy [51]. Moreover, the increased expression of zonula occludens-1 (ZO-1) tight junction proteins related to increased pericyte coverage highlights the role of pericytes in tight junction stabilization in developing retinal vessels within the first three postnatal weeks [52]. This evidence confirms the central role of pericytes in BBB and BRB structures, as well as in responding to metabolic demands.

### 2.4. Mesenchymal Stem Cell Features

As discussed above, the role of pericytes is not restricted to regulating and maintaining vasculature, but they are also able to differentiate into phagocytes, chondrocytes, adipocytes, myocytes, and osteoblasts [53,54,55]. For this reason, they have been identified as MSCs associated with microvasculature. Pericyte marker expression and morphology varies between different tissues and, as concerns morphology, they vary depending on the tissue, vessel size, or if they are active or quiescent [56]. 

The most common markers used to define pericytes include neural glial antigen 2 (NG2), PDGFR-β, CD13, CD146, 3G5 ganglioside, desmin, alkaline phosphatase, and αSMA. In particular, NG2 and αSMA expression correlates with the type of vessel they surround. Thus, pericytes that wrap capillaries are NG2^+^/αSMA^−^, those on venules are NG2^−^/αSMA^+^, and those on arterioles are NG2^+^/αSMA^+^, whereas PDGFR-β and CD146 are present everywhere [57].

The fact that MSCs have a perivascular origin has been demonstrated by the expression of the surface molecules commonly used as MSC markers, CD44, CD73, CD90, and CD105, in perivascular cells that are natively expressed. Moreover, human perivascular cells from diverse human tissues cultured for a long period give rise to multilineage progenitor cells, thus exhibiting MSC features [57]. Several markers in both pericytes and MSCs have been reported [58], showing the correlation between MSCs and pericytes. From this point of view, in our opinion and that of other researchers, microvascular resident pericytes could be considered a source of MSCs, able to mobilize following injury and/or disease, to exert their MSC potential.

Two models of MSC sources have been described. In the “lineage model”, specific MSCs could derive from tissue-specific pericytes, whereas in the “niche model” common MSCs originate from pericytes and acquire specific characteristics in response to the environment [59].

Pericytes are able to act as phagocytes, because they can absorb molecules by pinocytosis from extracellular fluid in the BBB and express features such as scavenging and Fc receptors [60]. Moreover, after incubation with chondrogenic medium, pericytes express type II collagen, aggrecan and the chondrocyte marker Sox-9 [54]. In response to thermal injury or when placed in adipogenic medium, pericytes convert into immature adipocytes [55] and express the adipocyte transcription factor peroxisome proliferator-activated receptor γ2. Furthermore, pericytes are able to convert into smooth muscle cells and it is noteworthy that also smooth muscle cells can convert to pericytes [61]. 

Pericytes, after the binding of macrophage-derived amphiregulin to the epidermal growth factor (EGF)-receptor, release bioactive TGF-β and can differentiate into collagen-producing myofibroblasts, which critically contribute to the restoration of vascular barrier integrity, thereby causing rapid tissue revascularization [62]. 

Last but not least, several studies showed that pericytes can also differentiate into neural cells, although they are not mesenchymal cell types [63].

The evidence so far reported on pericyte plasticity highlights their ability to represent a source of stem cells and lays the foundation for their future therapeutic use.

## 3. Pericytes in Brain Disease, Repair and Preservation

Neurological diseases such as ischemic stroke, traumatic brain injury, Alzheimer’s Disease (AD), amyotrophic lateral sclerosis and Parkinson’s disease are characterized by pericyte dysfunction [64]. Hereafter, short description of pericyte involvement in ischemia-reperfusion and brain tumor is presented.

### 3.1. Ischemia-Reperfusion

Stroke is caused by the obstruction of a blood vessel with the temporary arrest of the blood flow and consequent damage, often irreversible, of areas of nervous tissue [65]. In this scenario, the recanalization of the obstructed vessel represents effective treatment. In nervous tissue, the effects of recanalization depend on the functions of the cells that compose the neurovascular unit (NVU). As a response to stroke and the consequent hypoxia, pericytes relax, causing a temporary dilation of the capillaries in order to restore blood flow and nutritional intake [66]. Unlike the brain, cardiac pericytes narrow the capillary diameter after ischemia, often leading to a prolonged reduction in microvascular perfusion even after the restoration of blood flow (not reflux). Pericytes, responsible for this condition, represent a pharmacological target for the treatment of coronary no-reflow [67].

Several molecules control the shrinkage of pericytes during ischemia-reperfusion. It is established that adenosine accumulates significantly in the ischemic brain, acting as a vasodilator. Physiologically, high affinity A1 and low affinity A2a adenosine receptors activate ATP-sensitive potassium channels (KATP), whose opening results in hyperpolarization of pericytes and the dilatation of microvessels. This event would be exacerbated during the ischemic episode, causing a significant increase in microvascular dilation [68]. 

The level of PDGF-B increases during hypoperfusion and ischemia, and it has been reported that PDGF-B relaxes pericytes in order to increase microvessel diameter and blood supply [69]. Moreover, it has been reported that the amount of PDGFR-β^+^ cells is elevated in acute stroke [70]. In response to ischemia, the increased expression of bFGF in pericytes leads to the up-regulation of PDGFR-β. The PDGF/PDGFR-β axis is responsible for pericyte migration towards the newly formed BBB microvessels, and this event is needed for the maturation of microcirculation [69]. PDGF-B/PDGFR-β signaling restores ischemic tissue through pericyte recruitment [71]. 

**Figure 1:**
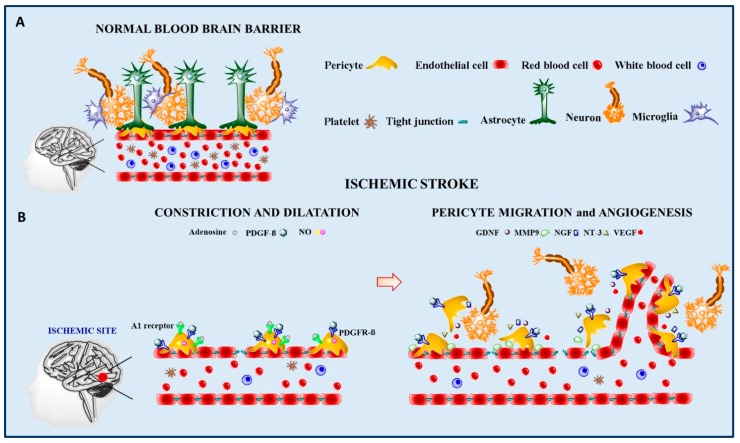
Schematic model of the blood brain barrier (BBB) in physiological conditions (**A**) and following ischemic damage (**B**). In the absence of any insult, the BBB is a highly organized structure, composed of different cellular types that cooperate to protect neurons from external agents circulating in the blood (**A**). In the presence of stroke, pericytes play a key role in restoring blood flow, by synthesizing different molecules involved in microvascular dilatation, new vessel formation and neuroprotection (**B**). A1 adenosine receptor (A1 receptor), adenosine, platelet-derived growth factor-B (PDGF-B), platelet-derived growth factor receptor-β (PDGFR-β), nitric oxide (NO), glial cell-derived neurotrophic factor (GDNF), matrix metallopeptidase 9 (MMP9), nerve growth factor (NGF), neurotrophin-3 (NT-3), and vascular endothelial growth factor (VEGF).

Following the release of endothelial PDGF, pericytes could exert an important effect on neuronal regeneration, expressing several neurotrophic factors, such as glial cell line-derived neurotrophic factor (GDNF), brain-derived neurotrophic factor (BDNF), nerve growth factor (NGF) and neurotrophin-3 (NT-3), with neuroprotective effects [72]. Pericyte-secreted NT-3 following ischemia could stimulate astrocytes to produce NGF, thus enhancing neuroprotection in the area close to the damage (Figure 1).

Pericytes secrete a large amount of Ang-1 and GDNF into the CNS, which are responsible for tight junction (TJ) maintenance. In vitro experiments performed with pericyte conditioned medium, under both normal and hypoxic conditions, revealed an increase of TJ proteins, ZO-1 and occluding expression in endothelial cells. Pretreatment with Ang-1 antibody upregulated this effect [73]. In in vitro BBB models, pericyte-secreted GDNF also up-regulated claudin-5 expression in EC and increased trans-endothelial electrical resistance (TEER) value [72].

Moreover, the secretion of large amounts of TGF-β enhanced VEGF receptor 1 (VEGFR-1) expression in endothelial cells, thus ensuring their survival in ischemic conditions [74]. 

The administration of VEGF in a middle cerebral arterial occlusion model of stroke decreased the necrotic areas, caused the up-regulation of pericyte coverage of microvessels and increased capillary density [75].

As is well known, the greatest damage to injured ischemic tissue is following tissue reperfusion, when pericytes undergo contraction. The influx of intracellular calcium and oxidative stress in pericytes occur after stroke [76]. In cultured retinal pericytes exposed to a free radical generating system, the translocation from the cytosol to the cytoskeleton of the myosin heavy chain, causing pericyte contraction, has been demonstrated [77].

In brain endothelial cells and pericytes, nitrosative and oxidative stresses are the result of the high expression of pro-oxidant NADPH oxidases [78]. Being free radicals and oxidative stress mediators of strong toxicity in pericytes in post-ischemic reperfusion damage, anti-oxidant molecules such as edaravone have been shown to promote pericyte proliferation and to maintain the structure and function of the neurovascular unit [79]. In this regard, edaravone reduces the production of metalloproteinase 9 (MMP9) by pericytes, thus attenuating BBB destruction during reperfusion injury [80].

In the middle cerebral arterial occlusion model of stroke in rats, pericytes move away from the basal lamina and migrate toward the hypoperfusion lesion [81]. Moreover, in ischemic conditions, pericytes release MMP9, thus interrupting the tight junctions between endothelial cells and themselves, increasing BBB opening and permeability [82]. In this scenario, pericytes migrate, drive angiogenesis in the hypoxic sites and could repair the NVU [83]. Evidence shows that hypothermia is protective for ischemic stroke because it retards pericyte migration from microvessels [84], ensuring the presence of pericytes on site, so as to be able to exert their neuroprotective effect, ensuring their increased secretion of NGF, NT-3 [85] and GDNF [72] following cerebral ischemia in the NVU. 

Because the hypoperfusion that results from vascular occlusion leads to necrosis of downstream tissue, the restoration of blood flow and oxygen supply is the solution to this damage [86]. The angiogenesis process is essential for the recovery of tissue function. It consists of the proliferation of endothelial cells, the recruitment of pericytes that cover the draft of the endothelial tube and, finally, the maturation of the new vessel. It has been shown that VEGF production by pericytes and the upregulation of fms-related tyrosine kinase 1(FLT1) expression increase during hypoxia [87,88]. The binding of VEGF to FLT1 results in a late proliferation of pericytes several days after hypoxia [88]. Moreover, VEGF induces the up-regulation of Ang-2 expression in endothelial cells and Ang-1 and its receptor Tie-2 in pericytes, which mediate further angiogenesis [89]. 

During neovascularization, the expression of the angiogenetic marker of regulator of G-protein signaling-5 (RGS-5) is elevated and depends on hypoxia inducible factor-1α (HIF-1α) levels. It inhibits pericyte proliferation and leads to pericyte maturation [90].

Moreover, pericytes are fundamental for leukocyte trafficking into the inflamed brain by regulating leukocyte adhesion and migration towards hypoxic tissue, an event that mediates the inflammatory responses that accompany ischemia [91]. Leukocyte migration through gap junctions (enlarged during inflammatory stimulus) between pericytes is mediated by the interaction between the intracellular adhesion molecule-1 (ICAM-1) of perycites with its integrin ligands on leukocytes [92].

In mice with focal brain ischemia, pericytes migrate into the injured brain parenchyma, become active and express markers of microglia (CD11b, IBA-1, GAL-1) after one week. In agreement with these observations, human pericytes under hypoxic conditions upregulate in vitro microglial genes (*GAL-2, CD11b, MHC11, IBA1, TNF-α*), further confirming the ability of pericytes to acquire microglia features [81].

Pericytes could exert an important effect on neuronal regeneration, expressing GDNF, BDNF [72], NGF, and NT-3 when endothelial PDGF increases [69]. 

### 3.2. Pericyte Regenerative Potential in Ischemia-Reperfusion

Because pericytes mediate pathological and repair processes in stroke, the direct pericyte graft into ischemic areas could represent a promising future strategy to promote tissue survival and NVU maintenance and regeneration, even if the BBB may constitute a “block” for the penetration of pericytes into the damaged brain. For this purpose, mannitol has been used to enable the crossing of pericytes to ischemic areas [93]. The beneficial effect of pericyte transplantation in ischemic areas has not been reported yet.

In vitro mouse and human pericytes, when cultured in oxygen/glucose-deprived media, reveal mesenchymal multi-lineage developmental properties because they differentiate in vascular and glial cells [94], and, by transducing the neurogenic transcription factors sox2 and ascl1, they could be reprogrammed into neurons [95]. In vivo, pericytes differentiate into glial cells rather than a neuronal lineage [96].

Novel therapeutic approaches to neurological disorders, focused on pericytes, are emerging. It has been shown that pericytes, administered in regions of the brain where damage has occurred, could improve the repair process [97]. 

It has recently been shown that brain-specific pericyte-like cells from induced pluripotent stem cells from humans displayed the stable expression of pericyte markers and are capable of inducing endothelial barrier properties and tube formation, providing the possibility to create autologous and “personalized” pericyte cellular therapies for brain neurodegeneration and diseases, avoiding the complications of immune rejection of cells [98,99].

### 3.3. Tumor 

In the brain, the pericyte/endothelial cell ratio is higher than in other tissues and for this reason, in the last few years, research has focused on the role of pericytes on the stabilization and progression of tumors, including gliomas and glioblastomas. Microvessels become abnormal in brain tumors because of their modification in structure, function and physiology, mainly as a consequence of the breakdown of existing blood vessels [100,101]. Tumor development is the consequence of the assessment of disorganized vascular networks, abnormal branchings, and lack of pericytes, all events which favor aberrant angiogenesis and metastasis [102]. 

Pericytes are supposed to be active cell components of tumor perivascular niches in glioblastoma, together with tumor cells, normal and reactive astrocytes, microglia/macrophages, myeloid cells, fibroblasts, and normal neural stem cells [103,104]. It has been demonstrated that pericytes, supporting newly formed blood vessel and tumor progression, derive from glioblastoma stem cells through trans-differentiation. In glioblastoma perivascular niches, pericytes undergo mesenchymal differentiation, thus supporting tumor growth and actively remodeling perivascular niches. These derived pericytes exhibit tumor-specific genetic alterations that allow fir discrimination between them and normal pericytes, indicating a possibility to selectively target these neoplastic pericytes [105].

The expression of anti-inflammatory molecules, such as IL-10, TGF-β, and MHC-II, is increased in glioblastoma-activated pericytes, thus favoring immunosuppression and tumor growth [106]. To date, it still remains to be elucidated what the other molecules (growth factors and/or cytokines) produced by brain pericytes supporting the tumor growth are. It would be interesting to investigate the possible origin of pericytes that transform into glioblastoma cells. Indeed, according to Stout and Murray’s hypothesis, it has been supposed that Zimmerman’s pericytes have a high “tumorigenic potential”, giving rise to hemangiopericytomas [107] that are histomorphologically similar to fibrous tumors [108].

Although phenotypically similar, the normal BBB and tumor-BBB differ. The latter is characterized by abnormal and highly proliferative microvessels [109], with altered physical contacts with endothelial cells and astrocytes [110]. The selective inhibition of BMX (a member of the Tec tyrosine kinase family), significantly expressed in glioma stem cell-derived pericytes, selectively affects the tumor-BBB (but not the BBB), allowing drug effusion into tumors and enhancing the chemotherapeutic efficacy of drugs [111]. These experimental approaches suggest the possibility to disrupt the blood–tumor barrier without affecting the normal vasculature in the neuropil, which is necessary for anti-cancer drugs to properly reach the affected tissue. As a consequence of this, targeting glioma stem cell-derived pericytes through ibrutinib (BMX inhibition, food and drug administration (FDA)-approved) could disrupt the blood tumor barrier but not the normal BBB. This therapeutic approach could synergize with the canonic therapies to approach patients affected by glioblastoma or other malignant brain tumors or metastases [111].

Aberrant tumor vessels are fenestrated with poor pericyte coverage, and this does not allow the chemotherapeutic molecule to reach the targeted tumor site. The question that remains is: Can there be a correlation between poor pericyte coverage of tumor vasculature with increased tumor growth and, moreover, metastatic spreading? In this regard, it has been reported that the loss of pericytes leads to an unexpected increase in tumor growth in mouse models [112].

### 3.4. Pericyte-Targeted Anti-Tumor Therapy

Microvessel normalization appears to be a temporary result of antiangiogenic therapy [113] and this event depends on the covering of pericytes. In pancreatic tumor-bearing mice, the deficiency of pericyte marker G-protein signaling 5 (*Rgs5*, a gene responsible for the aberrant tumor vasculature in mice), results in pericyte maturation, vascular normalization, and a marked reduction of hypoxia. Moreover, these actions enhanced the inflow of immune cells into the tumor, thus markedly prolonging the survival of tumor-bearing mice [114]. The microvascular normalization in cancer increases oxygenation, which makes radiation more effective against tumor cells, preventing metastasis, increasing the delivery of the targeted agents or chemotherapy, the efficacy of surgery or radiation, and recognition by the host immune system [115]. From this point of view, pericyte-targeted therapies (i.e., targeting the receptors for PDGFR-β, Tie-2, and VEGF) have the aim of reaching tumor vascular normalization.

It has been discussed that the association of an antitumor with an anti-angiogenic drug in a certain window of time (early angiogenesis) may restore the imbalance between pro- and anti-angiogenic factors, leading to the normalization of microvessels, and this could allow the chemotherapeutic drugs to reach the tumor [11,116]. In highly vascularized tumors, the inhibition of pericyte dependent-new angiogenesis may be a therapeutic target to prevent tumor growth and metastases. The block of PDGFR-β phosphorylation by imatinib inhibits tumor growth [117].

Glioma cells attract pericytes to newly formed microvessels by secreting PDGF-B and PDGFR-β signaling promotes IL-33 expression by pericytes. This triggers the recruitment of tumor-associated macrophages that promote tumor invasion [118]. Preventing IL-33 production, by targeting pericytes, could thus be used to slow down tumor growth.

Emerging physical strategies have been improved to ensure drug delivery across the BBB and tumor-BBB, this is a difficult event because barriers allow only the selective passage of small molecules. Docetaxel-conjugated nanoparticle delivery has been highlighted in pericytes from neuroblastoma rats [119] and a recent study has shown that the intranasal administration of plasmid DNA, encoding GDNF encapsulated within nanoparticles, was able to transfect perivascular cells, probably pericytes [120]. The delivery of drugs targeting pericytes carried by nanoparticles could provide novel therapeutic strategies for the treatment of many neurovascular diseases, including brain cancers.

## 4. Pericytes in Retinal Diseases and Repair 

Pericytes are particularly abundant in retinal microvessels [121]. It is well known that pericyte loss and BRB breakdown play a central role in the pathological development of age-related macular degeneration, uveitis, and diabetic retinopathy (DR) [122]. 

### 4.1. Diabetic Retinopathy

DR is the major complication of diabetes mellitus and is characterized by microaneurysms, vascular occlusion and neuronal suffering that ultimately lead to diabetic macular edema, proliferative DR and loss of vision [123]. 

Since the loss of pericytes represents the focal point of DR pathogenesis, the repositioning of these cells in the empty dimples could represent an effective treatment of this pathology. 

Stem cells have been used for the treatment of numerous diseases that affect different organs, including the eye. In particular, they have been used to give rise to photoreceptors, glia cells and neurons [124]. Studies on stem cells capable of reconstituting the retinal microcapillaries are numerically far inferior and, in recent years, the ability of pluripotent cells to re-establish the physiological contacts between microcapillary cells and neurons is emerging [125]. Moreover, intravitreal injection of endothelial progenitor cells has been able to repair retinal vascular damage in a mouse diabetic model [126].

The hypothesis that ocular pericytes have mesenchymal progenitor characteristics was formulated for the first time in the 1990s, demonstrating that retinal pericytes, grown to confluency, formed multi-layers containing multicellular nodules and hydroxyapatite, indicating their osteoblast characteristics [127]. 

For this reason, ocular pericytes play a role not only in ocular vessel stabilization, but also as a stem cell niche that contributes to regenerative processes [128]. 

In particular, a study on ocular diseases recently demonstrated that retinal pericytes can differentiate to adipocytes after in vitro treatment with the free amino acids hydroxyproline, lysine and glycine, which are elevated in the vitreous of proliferative diabetic retinopathy (PDR). The adipogenic effect in retinal pericytes induces a protective function by decreasing angiogenesis markers that are potentially pathogenic, and by increasing antioxidant response [129]. Retinal pericytes can also differentiate into chondrocytes, not only in in vitro conditions but also after in vivo transplantation [54]. 

In vivo and in vitro experiments have demonstrated that pericyte-expressed Tie-2 contributes to vessel formation and maturation. After pericyte-specific deletion of Tie-2, the amount of pericytes covering the retinal microvessel is strongly reduced, leading to delayed retinal vascularization [130].

**Figure 2:**
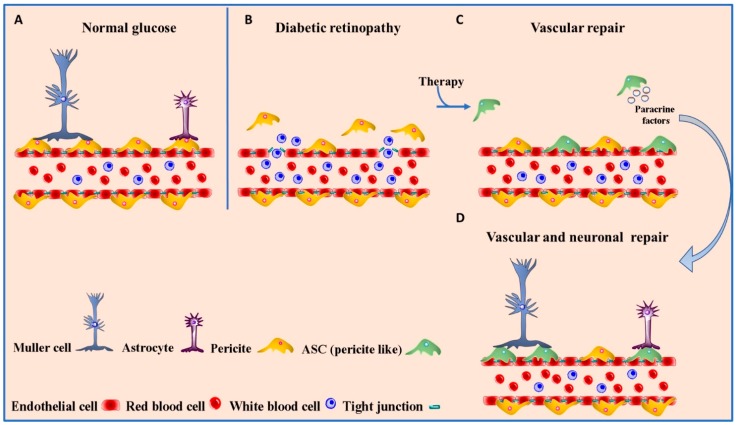
Schematic representation of the human blood retinal barrier (BRB) in the presence of normal glucose levels (**A**), in pathological conditions (**B**), under therapy with ASCs (pericyte like cells) (**C**,**D**). In the physiological state, specialized endothelial cells, connected by tight junctions, pericytes, Muller cells and astrocytes prevent the passage of potentially toxic molecules from the blood to the retina (**A**). Prolonged high glucose levels breakdown the BRB (**B**). The treatment with ASCs is able to make vascular and neuronal repair (**C**,**D**).

The involvement of inflammation in the pathogenesis of DR has been shown by numerous studies that have also highlighted a profound alteration of the neuro-sensory retina as well as vascular perturbations [131]. For this reason, potential cell therapies should aim to insert cellular elements capable of having the dual function of stabilizing the vessel and, at the same time, freeing molecules with an anti-inflammatory effect (Figure 2).

The ability of pericytes to differentiate into adipocytes leads us to reflect on the link between the two cell types. Indeed, the inverse relationship between the two cell types has also been demonstrated, as adipose stromal cells have the extraordinary property of becoming pericytes, expressing identical surface markers PDGFR-β, NG2 and N-cadherin [132]. These results make these cells attractive candidates for the treatment of DR.

Previous studies demonstrated that the intravitreal injection of bone marrow-derived stem cells (BMSCs) was able to repair retinopathic insults by transforming into photoreceptors, microglia and endothelial cells but not into pericytes [132]. On the other hand, adipose-derived stem cells (ASCs) can differentiate into pericytes, when injected intravitreally, thereby integrating with the retinal microvasculature. In these conditions ASC-derived pericytes adopt pericyte morphology, showing pericyte-specific markers, and their phenotype is enhanced with TGF-β1 treatment demonstrating that ASCs could be used to protect against DR and to treat retinal vascular disease. [133]. Due to the unavailability of human retinal pericytes for transplantation, these cells, that are able to stabilize existing retinal microvasculature, could therefore represent an alternative therapy to the destructive laser treatment of hypoxic retina. Moreover, it has been demonstrated that ASCs, which remain in hypoxic conditions in adipose tissue, which is greater than that of the eye, are able to survive after transplantation [134].

The delivery of ASCs has been studied to prolong their lifespan in a model of diabetic wound regeneration, demonstrating that, in diabetic rats, encapsulated ASCs in in situ crosslinked hydrogels are able to accelerate wound closure [135]. Future translational applications could be encapsulated ASCs injected into the vitreous, which could be a promising potential therapy to stabilize retinal vessels, and to restore the neurovascular unit by releasing trophic factors into the microenvironment without direct contact with microvascular endothelial cells.

Conversely, it would be appropriate to reflect on the fact that ASCs could be affected by diabetes, which alters the adipose tissue metabolism and this condition would implicate their possible failure to induce beneficial effects in retinopathy. Some studies reported similar functions and capabilities, whereas others identified differences between healthy and diabetic donors [136]. In particular, in vivo experiments demonstrated that implanted diabetic ASCs in healthy mice failed to induce new vascularization in comparison with healthy ASCs [137].

Summing up what has been stated above, the research on advanced therapies for the treatment of DR is making great strides and represents a promising future for the many patients suffering from this invalidating pathology.

### 4.2. Uveal Melanoma

Pathological neovascularization also occurs in uveal melanoma and it has been suggested that pericytes play a crucial role in forming new vessels. It has been demonstrated that the inhibition of pericyte-NG2 proteoglycan decreases neovascularization and tumor volume, rendering pericytes and NG2 proteoglycan as potential cellular and molecular therapeutic targets in orthotopic uveal melanoma [138].

There are very few studies in this field and those that are date back to more than ten years ago [139] because microvascular endothelial cells in melanoma have been studied extensively whereas much less is known about pericytes. It has been demonstrated that retinal pericytes are associated with microvessels before BRB tight junction expression, whereas the important prerequisite for the genesis of the barrier in the brain is pericyte recruitment [140]. 

Recently, the role of pericytes in the target organ microenvironment during melanoma cell extravasation has been investigated. In particular, in PDGF-B^ret/ret^ transgenic mice (PDGF-B mutant), characterized by microvessels with defective pericyte coverage, no invasion by melanoma cells in bone marrow and liver was observed, demonstrating that the presence of pericytes is required for the extravasation of melanoma cells [141].

Moreover, VEGF is more effective for survival of vessels with low pericyte coverage than for vessels with high coverage of pericytes, suggesting that tumors with a propensity to recruit pericytes are particularly resistant to antiangiogenic therapies. This condition makes pericytes a relevant target for anti-tumor therapy. For this reason, the understanding of the molecular mechanism involved in pericyte recruitment, as well as PDGF-B production, could predict the outcome of antiangiogenic therapy [12].

A recent study demonstrated that antiangiogenic agents, such as vascular disrupting agents (VDA), failed to affect the most peripheral vessels, which are particularly rich in pericytes, indicating that pericytes might be closely related to VDA treatment resistance. In the light of this aspect, the study demonstrated the efficacy of a derivative of vinblastine, which acts selectively towards pericytes, inducing a complete regression of multiple lines of tumor xenografts and VDA treatment resistance [142]. The inhibition of PDGF-B signaling by an anti-PDGFR antibody causes destabilization of the retinal vasculature, suggesting that pericyte recruitment and their interaction with endothelial cells play an important role in the pathological angiogenesis of ocular disease. For these reasons, to address the problem of resistance to cancer therapy, a combination therapy approach, using both VEGF-A and PDGF-B inhibitors, could be a more effective intervention for multiple models of ocular neovascularization [143]. 

## 5. Conclusions and Future Perspectives

Pericytes play a pivotal role in the development and formation of the vascular network as well as in tissue homeostasis. However, increasing interest in this type of cell comes from recent evidence of their MSC features. In order to pursue the goal of replacing or regenerating damaged or exhausted tissue through the control of the function and differentiation of MSCs and pericytes, there are several issues that should be addressed. For example, how can stem cells be integrated into a tissue? What will the immunomodulatory effect of these cells at the injured site be? How will it be possible to determine the most useful population of stem cells for the regeneration of a given tissue? Most importantly, the translation of data from in vivo and in vitro studies on humans will be problematic, and there will be rigorous controls both during clinical trials and at post-treatment patient follow-ups in order to gain a consensus on the therapeutic efficacy of pericyte/MSC therapies.
